# Reverse vaccinology for hookworms: a rational selection of vaccinable antigens against parasitic nematodes

**DOI:** 10.1051/parasite/2025025

**Published:** 2025-06-03

**Authors:** Javier Sotillo, Mónica Quinzo, Juan José García, Antonio J. Martín-Galiano

**Affiliations:** 1 Laboratorio de Referencia e Investigación en Parasitología, Centro Nacional de Microbiología, Instituto de Salud Carlos III Ctra Pozuelo km. 2 28220 Majadahonda Spain; 2 Escuela Internacional de Doctorado, Universidad Nacional de Educación a Distancia (UNED) 28015 Madrid Spain; 3 Departamento de Microbiología y Parasitología Facultad de Farmacia, Universidad Complutense Plaza de Ramón y Cajal, s/n 28040 Madrid Spain; 4 Core Scientific and Technical Units, Instituto de Salud Carlos III Ctra Pozuelo km. 2 28220 Majadahonda Spain

**Keywords:** Antigen, Epitope, Reverse vaccinology, Parasites, Nematodes, Immunoinformatics, Hookworms, Vaccine development

## Abstract

Reverse vaccinology is a time- and cost-effective approach to identify potential vaccinable antigens for further *in vivo* experimental validation. Despite its wide application to multiple organisms, the use of *in silico* vaccine development tools to parasitic nematodes has been limited. Herein, we have used the rodent hookworm *Nippostrongylus brasiliensis* as a mouse model for the human hookworm *Necator americanus* to identify potential vaccine candidates against the latter. Our strategy combined advanced bioinformatic evaluations with knowledge-based criteria. A cumulative rating of antigenic properties was performed resulting in a global prioritization scoring for an updated *N. brasiliensis* proteome of 22,796 proteins assigned. Evaluation criteria included homology to the human counterpart *N. americanus*, absence of mammalian homologs, cellular location by computational predictors, as well as mass spectrometry data, proteolytic activity of the evaluated protein within the parasite, presence of conserved domains, predicted humoral epitopes, and MHC class II epitope population coverage. To assign one global score representing these characteristics, cumulative scoring was performed. This analysis provided a group of 56 potential candidates, including 11 proteins associated with parasite survival and establishment. Remarkably, the second highest score was assigned to an aspartic protease homologous of the *N. americanus* vaccine-candidate *Na*-APR-1, which supports the relevance of this approach. Allergenicity and toxicity of the selected molecules were also predicted to anticipate side effects of future candidates. This comprehensive approach provides valuable insights for the rational design of new vaccines against *N. americanus*, the results of which, however, must be validated *in vivo*.

## Abstract (fr)

La vaccinologie inverse est une approche rapide et économique permettant d’identifier des antigènes vaccinables potentiels en vue d’une validation expérimentale *in vivo*. Malgré sa large application à de nombreux organismes, l’utilisation des outils de développement de vaccins *in silico* aux nématodes parasites est limitée. Dans cette étude, nous avons utilisé l’ankylostome de rongeur *Nippostrongylus brasiliensis* comme modèle murin de l’ankylostome humain *Necator americanus* afin d’identifier des candidats vaccins potentiels contre ce dernier. Notre stratégie a combiné des évaluations bioinformatiques avancées avec des critères fondés sur les connaissances. Une évaluation cumulative des propriétés antigéniques a été réalisée, ce qui a permis d’établir un score de priorité global pour un protéome actualisé de *N. brasiliensis* comprenant 22 796 protéines. Les critères d’évaluation comprenaient l’homologie avec son homologue humain *N. americanus*, l’absence d’homologues mammifères, la localisation cellulaire par des prédicteurs informatiques ainsi que des données de spectrométrie de masse, l’activité protéolytique de la protéine évaluée au sein du parasite, la présence de domaines conservés, les épitopes humoraux prédits et la couverture de la population d’épitopes du CMH de classe II. Afin d’attribuer un score global représentant ces caractéristiques, un score cumulatif a été calculé. Cette analyse a fourni un groupe de 56 candidats potentiels, dont 11 protéines associées à la survie et à l’établissement du parasite. Fait remarquable, le deuxième score le plus élevé a été attribué à une protéase aspartique homologue du candidat vaccin contre *N. americanus*, Na-APR-1, ce qui confirme la pertinence de cette approche. L’allergénicité et la toxicité des molécules sélectionnées ont également été prédites afin d’anticiper les effets secondaires des futurs candidates. Cette approche globale fournit des informations précieuses pour la conception rationnelle de nouveaux vaccins contre *N. americanus*, dont les résultats doivent cependant être validés *in vivo*.

## Introduction

Soil-transmitted helminths infect more than 1.5 billion people worldwide, particularly in low-income countries with inadequate sanitation and hygiene practices [43, 62]. Approximately 50% of these infections are caused by *Necator americanus* and other hookworms. These blood-feeding parasitic nematodes cause anemia, dysentery, impaired cognitive development, and in combination with malnutrition, increase susceptibility to other diseases [15, 58]. Furthermore, given the high burden of hookworm infections and their impact on global public health, the World Health Organization has included the development of effective control measures against this group of parasites in its 2030 priority goals [62].

Despite the availability of generic anti-helminthic drugs against hookworms, their use presents several disadvantages. For instance, they do not prevent reinfection nor provide long-term protection [21]. In addition, the difficulty associated with treating mass populations and the emergence of drug resistance support the development of vaccines to control this devastating disease. Following the successful use of attenuated worms and recombinant proteins in animal models, human clinical trials have been carried out [6]. However, the first human vaccine studies demonstrated limitations, such as generalized urticaria or reduced antibody responses [61]. To overcome these challenges, further research has been conducted to identify crucial proteins for parasite survival and establishment, such as those involved in blood-feeding and heme detoxification [60]. Two proteins that are highly expressed in the gut of hookworms, *Na*-APR-1 and *Na*-GST-1, have been tested for human vaccination in two clinical trials in adults and children from Gabon [1, 65]. Adult vaccination was considered safe and sufficient to induce specific IgG responses; thus, phase II clinical trials are ongoing [11]. For children, early results of a phase I clinical trial confirmed safety, but further details about immunization responses have not yet been released [61].

Extensive research aimed at developing a human hookworm vaccine has been conducted using mouse models, facing various challenges such as extended life cycles, reduced availability of experimental worms, and lack of culture protocols for all life stages [38]. Indeed, research on *N. americanus* is hindered by the inability to maintain this parasite in rodents. To overcome these limitations, *Nippostrongylus brasiliensis* is commonly utilized as a model to study human hookworm infections. This is due to the morphological, developmental, and proteomic similarities between this rodent nematode and *N americanus*, as well as their comparable hematophagous lifestyle [38]. *Nippostrongylus brasiliensis* not only induces a typical Th2 type immune response that manifests all the characteristics of a human hookworm infection [39], but also shares high sequence identity between homologs and similar proteome content with *N. americanus* [38]. A remarkable parallelism has been identified between the secretomes of both nematodes at different developmental stages [33, 54]. For instance, *N. brasiliensis* contains homologs of recombinant antigens investigated for human hookworm vaccination and proteins associated with hookworm immune evasion [4, 56]. Indeed, immunization of mice with *Na*-APR-1 has been shown to provide cross-protection against *N. brasiliensis* challenge [3].

Traditional hookworm vaccines rely on the production of irradiated worms or isolation and purification of potential antigenic components of the parasite. This approach is not only time consuming and costly, but also presents challenges in identifying suitable antigens for vaccine development. Vaccine efficacy is influenced by different factors, including major histocompatibility complex (MHC) allelic restriction in the host and the diverse immune evasion strategies used by these parasites [61]. In recent years, reverse vaccinology approaches have been successful in identifying potential vaccine candidates for viruses and bacteria, among others [22]. These approaches have shown great promise in rationalizing the computational selection of candidates before undergoing further *in vitro* and *in vivo* experimental validations. For instance, this approach has been used for the development of vaccines against a wide range of bacteria, such as *Neisseria meningitidis*, Group B *Streptococcus*, *Porphyromonas gingivalis*, and *Pseudomonas aeruginosa*; and viruses, including the Enterovirus A71 and avian influenza A virus (H7N9) [44, 46, 50].

Novel high-throughput technologies have been helpful in identifying not only the genomes but also the transcriptomes and proteomes for relevant conditions of several parasitic helminths and protozoans. This has opened new avenues for the application of reverse vaccinology to human parasites that cause neglected tropical diseases, including the intracellular parasite *Leishmania donovani*, the trematodes *Schistosoma japonicum* and *Schistosoma mansoni,* and the nematodes *Strongyloides stercoralis, Wuchereria bancrofti, Ascaris lumbricoides,* and *Ascaris suum* [8–10, 13, 17, 18, 42, 64]. For instance, six epitopes from the *L. donovani* proteome showed immunogenic potential in activating Th1 and Th17 responses in mice splenocytes, which are essential for protective immunity [13]. Rational strategies have also been successfully applied to the trematode *S. japonicum* to identify five promiscuous protective T-cell epitopes [64], two of them validated *ex vivo* in mice as inducers of a Th1-type response. Oliveira *et al*. applied a similar strategy to *S. mansoni* and showed that two of the ten predicted immunogenic glycopeptides successfully bound to murine MHC class II molecules and stimulated CD4+ T cell proliferation [10, 42].

The application of reverse vaccinology has recently also retrieved valuable data for vaccine design against human parasitic nematodes. For instance, the computational analysis of the proteomes of *S. stercoralis* and *Ascaris* spp. revealed 34 and 4 promising immunogenic candidates, respectively, suitable for novel vaccine design, but further validation and testing are necessary [8, 17]. Considering the success of *in silico* predictions of epitopic peptides for vaccine research and recently published works focusing on the *in silico* design of novel vaccine candidates against parasitic nematodes [9, 17, 18], herein we present the first reverse vaccinology approach applied to hookworms. Combining state-of-the-art bioinformatic analyses with a knowledge-based approach has enabled the evaluation of the hookworm proteome, and thus, the selection of potential vaccine candidates that can now be further tested for efficacy in both mice and humans.

## Materials and methods

**Figure 1:**
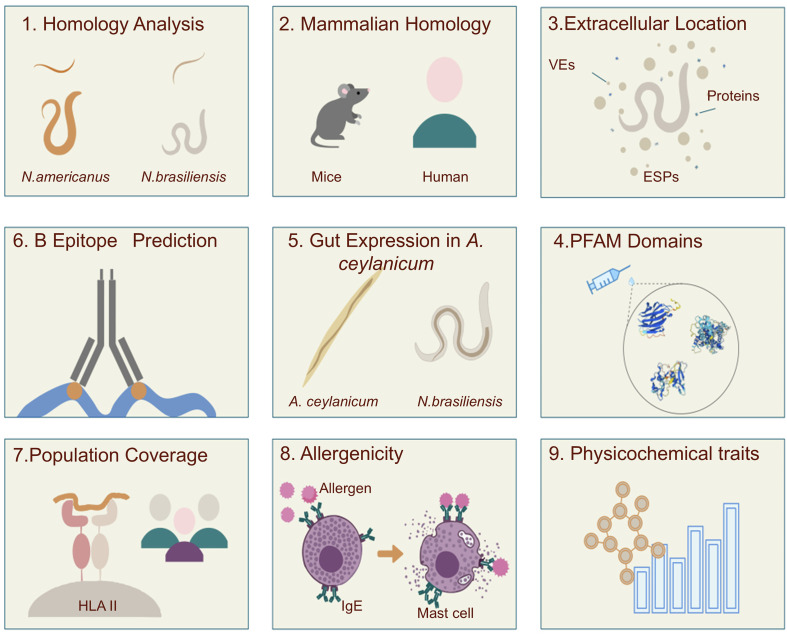
*In silico* vaccine discovery workflow aimed at identifying potential vaccinable antigens for the treatment of hookworm disease.

The global workflow is shown in Figure 1 and each analysis step is detailed below.

### Protein homology between hookworm species

The published draft genomes of *N. brasiliensis* and *N. americanus* contain 22,796 and 15,728 coding genes, respectively (https://parasite.wormbase.org/index.html) [26, 33]. To identify homologous proteins that may be involved in human and mouse hookworm infections, BLAST searches were performed to determine sequence identity between *N. brasiliensis* and *N. americanus*. Considering the lack of fully assembled genomes, we selected a minimum of 0.4% of length-aligned sequences as the cut-off for evaluating sequence identity [49, 57]. This cut-off is a balance between losing potential domains with small lengths and leaving behind sequences with low potential to be functionally defined. Scoring was based on the level of identity as follows: 5, 4, 3, 2, and 1 point for over 80%, 70%, 60%, 50%, and 40% of homology, respectively.

### Protein homology between hookworms and mammal species

To evaluate potential cross-reactivity with host proteins and thus select only those with the best likelihood of being safe and effective vaccine candidates, a comparative proteomic analysis between the predicted proteome of *N. brasiliensis* and the proteomes of *Mus musculus* and *Homo sapiens* was performed. BLAST searches were performed using the NCBI non-redundant (NR) collection of *M. musculus* and *H. sapiens* downloaded from UniProt (accessed 01 December 2022). A minimum length of 0.4% was used to score the aligned sequences. In this case, higher scores were given to proteins with lower levels of identity: 5, 4, 3, 2, and 1 for less than 20%, 30%, 40%, 50%, and 60% homology, respectively, for both mouse and human analysis. For the mammalian score, we selected the lower score between mouse and human homology for each protein.

### Extracellular location

We further narrowed down potential vaccine candidates by focusing on proteins that are known to be secreted or located in the extracellular space. The value of these proteins is based on the potential role of host-parasite interactions in infection, migration, immune evasion, and establishment of these parasites [41]. The classification of these proteins was based on previous proteomic analyses of the excreted and secreted (ES) products from L3 and adult *N. brasiliensis*, as well as data from extracellular vesicles secreted by adult worms [36, 54]. When analyzed, proteins were found in either one or two of the three possible locations. A score of ten was assigned to proteins located in two locations and five to those found in one location. Proteins without an identified location (scored 0) were further analyzed for the presence of signal peptides (SPs) at the N-terminal amino acid sequences using SignalP-6.0 with the “Eukarya” option [55]. SPs are short amino acid sequences that control protein secretion and translocation in all living organisms. Accordingly, proteins not identified in the proteomic analysis of ES products may still be expressed at lower levels in the extracellular space and can be identified using this novel machine learning model. Including these criteria, we scored these proteins as 3 or 0 when the signal peptide was detected or was not detected, respectively.

### Gut location

Proteins expressed in the gut are potentially involved in blood digestion and are, thus, crucial for the establishment of adult parasites in the host small intestine. Moreover, they are less likely to be exposed to the host immune system during previous natural infections and are, thus, less capable of generating urticaria and allergic reactions after vaccine administration. Based on previously published data from the intestinal transcriptome of the related hookworm *Ancylostoma ceylanicum* [60], we also carried out a homology analysis between *N. brasiliensis* and *A. ceylanicum* proteomes to identify proteins highly expressed in the gut. Only proteins with at least 60% sequence alignment and at least 30% homology with *A. ceylanicum* were evaluated. Considering these criteria, we classified the proteins into the following groups: top 100 proteins with the highest expression, proteins that were upregulated but not in the top set, proteins that were expressed in the gut but not upregulated, and those that were not detected in the gut. Accordingly, we assigned scores of 5, 3, 1, and 0 to these groups, respectively.

### Functional domain identification

Functional domains were identified based on those annotated in the PFAM database (https://www.ebi.ac.uk/interpro/entry/pfam/#table) [37]. The PFAM database classifies proteins into groups of related sequences and domains, allowing for the comparative analysis of undefined proteins to understand the underlying molecular biology of any organism. Protein scoring was based on a list of prioritized functions found in the vaccine candidates tested, including astacins, aspartic proteases, and cathepsins [35, 61]. Astacins, besides aspartyl- and papain cysteine-proteases, were classified into the top importance Group 1. Other cysteine and metallopeptidase family proteins were classified into the still relevant Group 2. The remaining peptidases and proteases were allocated to Group 3, whereas proteins with any other Pfam domain were allocated to Group 4. The scores assigned to these groups were 5, 3, 1, and 0, according to the level of prioritization described.

### Identification of linear B cell epitopes

Potential linear B-cell epitopes were predicted using BepiPred-3.0, available at (https://services.healthtech.dtu.dk/services/BepiPred-3.0/) [7]. BepiPred-3.0 is a sequence-based epitope prediction tool with improved accuracy for both linear and conformational epitope predictions based on protein-language models. When evaluating our proteins, a size of 9 amino acids was set for epitope length within a threshold of 0.1512 for predicting B-cell epitope residues. For each protein, BepiPred-3.0 identified several residues with the potential to be epitopic. We assigned a score to a given range of residues identified in each protein as follows: over 120, 90, 60, 30, 1, and 0 residues were scored as 5, 4, 3, 2, 1, and 0, respectively. In addition, the proportion of residues predicted to be epitopic over the entire sequence was determined.

### Population protection coverage

HLA II presentation is critical for eliciting T cell helper responses, particularly Th2, in the immune response to parasitic nematodes [2]. Population protection coverage can be determined by considering five HLA II supertypes that represent over 90% of the global population [23]. The affinities of all sequences were evaluated for four human HLAs: HLA-DR1, HLA-DR3, HLA-DR4, and HLA-DR [47]. The proteins were analyzed to predict epitopes with high affinity for these supertypes using the software NetMHCIIpan 4.0 (https://services.healthtech.dtu.dk/services/NetMHCIIpan-4.0/) [40, 48]. The NetMHCIIpan 4.0 program uses a maximum affinity score of 50, and any affinity values below this score were considered significant. We assigned increasing scores to proteins containing sequences predicted to have a high binding affinity to these HLA II supertypes. Thus, the score was given by the number of supertypes from zero to five.

### Allergenicity, toxicity, and physicochemical properties of top-15 candidates

After global analysis and scoring of the entire *N. brasiliensis* proteome, we selected the top 15 scoring proteins and their homologues in *N. americanus* for further evaluation, considering new parameters such as allergenicity, toxicity, and other physicochemical properties that can be considered for the experimental evaluation of these promising candidates. For each analysis, protein sequences were processed using the recommended settings for each server.

Three allergenicity algorithms, AllerTOP V.2.0, AlgPred, and SDAP 2.0, were selected to provide consistent data for a complete computational evaluation of allergenicity. AllerTOP v.2.0 is an alignment-free server for the *in silico* prediction of allergens based on amino acid sequences and physicochemical properties. Each protein was represented as a vector of 45 variables and compared to the AllerTOP set of food, inhalant, and toxin allergens (more than 2,400 proteins) from various databases and confirmed non-allergens [14]. AlgPred 2.0, similar to AllerTOP v.2.0, has been developed for predicting allergenic proteins as well as for mapping IgE epitopes in proteins. AlgPred predicts allergens based on their similarity to known allergens from a dataset of more than 10,000 allergens and more than 10,400 experimentally validated immunoglobulin E epitopes obtained from the IEDB database [53]. SDAP 2.0 is a web server that consists of a database and a computational component. The SDAP database contains information on allergens (name, source, and structure) and IgE epitopes. The SDAP 2.0 algorithm is based on conserved properties of the amino acid sequence to identify regions of known allergens similar to user-supplied peptides [27, 28, 51].

Additionally, we analyzed the potential human toxicity of the highest-scoring candidates using ToxinPred2 to further characterize their harmful potential. ToxinPred2 is a web-based tool developed to predict protein toxicity based on the SwissProt datasets. This tool combines three techniques for predicting protein toxicity to balance the quality of prediction with the sensitivity of the tool [52]. It combines local alignments with the identification of motifs and machine learning-based classifiers to analyze user proteins in comparison with toxins, such as bacterial toxins, neurotoxins, and venoms.

Finally, we complemented this analysis by evaluating the other physicochemical properties using ProtParam and SoluProt. ProtParam is a protein analysis tool that calculates the various physicochemical properties of a particular protein sequence using the SwissProt database. It is designed to provide information on protein composition, such as molecular weight, theoretical isoelectric point (pI), and amino acid composition [20]. SoluProt is a sequence-based prediction method for soluble protein expression in *Escherichia coli* that is used to help prioritize targets in large-scale proteomics projects [24]. It was developed using the gradient boosting machine technique with the TargetTrack database as the training set. Signal peptides, identified by SignalP 6.0, were removed prior to solubility analysis.

## Results

### Identification of *N. brasiliensis* proteins with homologs in *N. americanus* or mammals

**Figure 2:**
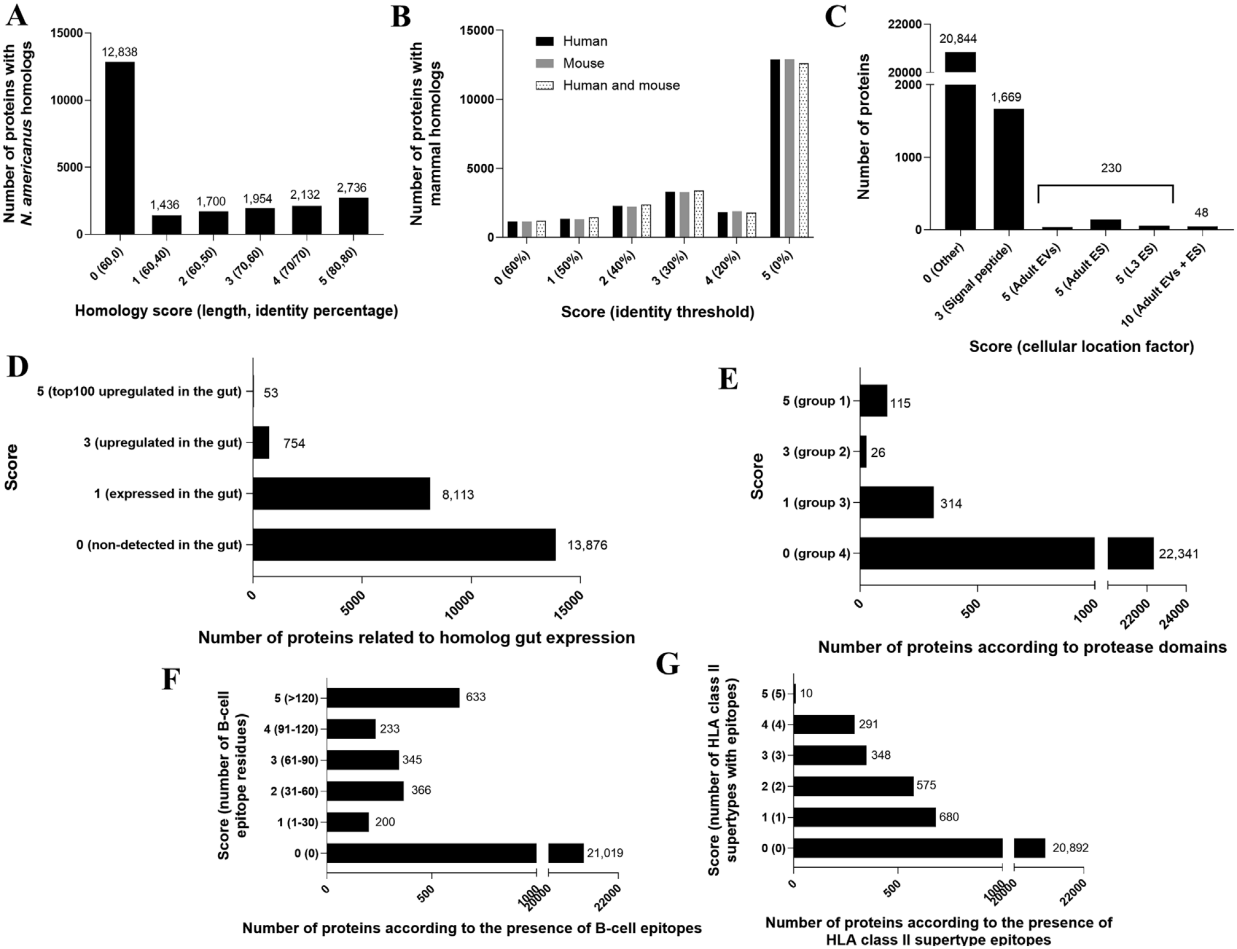
Number and scoring of *N. brasiliensis* proteins fulfilling the criteria of the applied reverse vaccinology approach. The figure presents the number of *N. brasiliensis* proteins: (A) with homologs in *N. americanus* based on different sequence length coverage thresholds (60%, 70%, or 80%) and identity threshold (0%, 40%, 50%, 60%, 70%, or 80%) combinations; (B) with homologs in *H. sapiens* and *M. musculus* reference proteomes at distinct identity cut-offs (0%–60% homology); (C) categorized by cellular locations of immunological interest; (D) with homologs expressed in the gut of the related hookworm *A. ceylanicum*; (E) with protease functional homologs (see Materials and methods for group details); (F) binned according to the number of residues within predicted B-cell epitopes; (G) binned according to the number of HLA class-II supertypes with at least one predicted epitope.

The initial evaluation of *N. brasiliensis* vaccine candidates included a sequence identity assessment with respect to its human counterpart, *N. americanus*, to select those showing the highest. This evaluation aimed to identify the proteins with the highest similarity to *N. americanus*, while simultaneously considering the absence of similarity with the host proteins to avoid potential immune cross-recognition of self-antigens. The identities calculated retrieved the distribution shown in Figure 2A and Supplementary Table 1. An identity below 40%, in combination with >60% alignment coverage length, was observed for 56.3% (12,838 proteins) of the *N. brasiliensis* predicted proteome. Among the proteins with the highest score, 2,736 *N. brasiliensis*-predicted proteins displayed over 80% identity and >80% alignment length.

Comparable analyses were carried out between the *N. brasiliensis* and mammalian proteomes, human and mouse, shown in Figure 2B and Supplementary Table 2. For both mammalian species, over 55% of the *N. brasiliensis-*predicted proteome (12,602) was considered non-homologous and assigned a score of five points.

### Location analysis

To enhance the robustness of our approach for identifying potential vaccine candidates, cellular locations appropriate for immune responses were considered. We assigned higher scores to proteins from the experimental secretome of *N. brasiliensis* previously identified by mass spectrometry, or those that harbored a predicted signal peptide (Fig. 2C, Supplementary Table 3). More than 8% of the *N. brasiliensis-*predicted proteome was located in the extracellular space: 48 proteins were found in two compartments, 230 proteins were found in only one compartment, and 1,669 contained a predicted signal peptide at the N-terminal of the sequence. Of those located in the described compartments, 77% were found in either excreted secreted products (ES) or extracellular vesicles (EVs) secreted by adult worms.

Two of the current vaccine candidates in clinical trials play a role in the digestion process of hookworms during the gut phase, and this feature was therefore considered. Since protein expression data in this key infection stage are neither available for *N. brasiliensis* nor *N. americanus*, a comparison of the *N. brasiliensis* proteome with the gene products identified and quantified in the gut of the related hookworm *A. ceylanicum* was performed (Fig. 2D, Supplementary Table 4). Nearly 40% of the *N. brasiliensis-*predicted proteome shared the required identity with proteins *of A. ceylanicum* produced in the host gut, from which 3.3% proteins involved homologs overexpressed in this niche. Furthermore, up to 53 *N. brasiliensis* proteins were homologs among the top 100 *A. ceylanicum* gut upregulated proteins and were assigned the highest possible score for this criterion.

### Crucial functions in parasite cycle

It is well established that proteases play a prominent role in hookworm pathogenesis [31]. Thus, and based on current knowledge, we stratified proteases into different scoring levels according to their potential role in infection. To this end, we designed a Pfam-based scheme to allocate *N. brasiliensis* proteins into four incremental groups according to the domain families to which they belonged (Fig. 2E, Supplementary Table 5). These domains were selected after exhaustive literature screening, as they are present in different vaccine candidates from helminths [31]. A total of 115 proteins contained proteolytic domains similar to those used as vaccine candidates in other helminths.

### Immunogenicity

The predicted antigenicity and immunogenicity were also considered. Proteins were prioritized by the raw number of predicted residues in B-cell epitopes, as well as the presence of HLA class-II epitopes with extended population coverage, defined as supertypes (Figs. 2F and 2G, Supplementary Tables 6 and 7). The former are involved in the humoral response and proved central to protection against nematodes, while the latter are part of the cellular response involving T helper lymphocytes and are dependent on individual genetics. A total of 21,019 and 20,892 proteins were predicted to lack B-cell or HLA class-II supertype epitopes, respectively, under our conditions. Of the immunogenic proteins, 633 were predicted to contain 120 or more B-cell epitopic residues and were given the highest score, whereas only 10 proteins were predicted to contain high-affinity epitopes present in all the five HLA class-II supertypes.

### Allergenicity, toxicity, and physicochemical properties of top-15 candidates

The combined score of the predicted *N. brasiliensis* proteome was distributed as shown in Supplementary Table 8. More than 20,000 predicted proteins scored lower than 10 and only 56 scored over 20. Table 1 displays a summary of the individual and combined scores of the top 15 candidates. These prioritized *N. brasiliensis* antigens were further screened for allergenicity (Table 2, Supplementary Table 9), toxicity, physicochemical properties, and solubility under standard laboratory conditions (Supplementary Table 10) for their practical utilization in vaccine formulations. Equivalent protein features were calculated for the *N. americanus* counterparts (Table 3 and Supplementary Tables 11 and 12). Only two top-scoring proteins (NBR_NBR_0000892601 and NBR_0001394301) from *N. brasiliensis* were found to be allergenic by the three programs applied, whereas none of the *N. americanus* homologues were allergenic by any of these algorithms. In contrast, only one candidate (NBR_0000928101) from *N. brasiliensis* was found to be non-allergenic in all results, while three *N. americanus* proteins (NAME_01959, NAME_03502, and NAME_05701) retrieved non-allergenic results in all analyses. Regarding toxicity, four *N. brasiliensis* (NBR_0001337201, NBR_0000897601, NBR_0001394301, NBR_0001356301) and two *N. americanus* (NAME_06878, NAME_13843) proteins were predicted to be toxic, respectively.

**Table 1:** Summary of all the results from each evaluation applied to the top 15 *Nippostrongylus brasiliensis* candidates ranked by total score. Sc_Nam: score obtained from the homology analysis against *Necator americanus*; Sc_Mammal: score obtained from the homology analysis against *Homo sapiens* and *Mus musculus*; Sc_locat: score obtained from the extracellular localization analysis, Sc_protease: score obtained from the functional analysis; Sc_Ace: score obtained from the homology with expression of gut proteins in *Ancylostoma ceylanicum*; Sc_Bepit: score obtained after B-cell epitope prediction analysis; Sc_MHCII: score obtained after MHC-II prediction analysis; Score: total score obtained for each protein.

Protein	Sc_Nam	Sc_Mammal	Sc_locat	Sc_protease	Sc_Ace	Sc_Bepit	Sc_MHCII	Score
NBR_0000584601	1	5	10	5	0	5	0	26
NBR_0000892601	5	1	10	5	1	2	1	25
NBR_0001337201	5	3	10	0	0	5	2	25
NBR_0000752401	3	5	5	0	5	5	1	24
NBR_0000897601	3	5	10	0	3	3	0	24
NBR_0001090101	5	3	5	5	1	3	2	24
NBR_0001394301	5	3	10	0	1	5	0	24
NBR_0000014101	3	5	10	0	1	4	0	23
NBR_0000612601	5	5	3	0	1	5	4	23
NBR_0000723701	3	4	5	1	1	5	4	23
NBR_0000844701	4	5	10	0	1	3	0	23
NBR_0000992501	3	4	10	1	1	2	2	23
NBR_0000823901	2	5	3	5	1	5	1	22
NBR_0000928101	4	5	3	0	5	5	0	22
NBR_0001356301	1	5	10	0	1	5	0	22

**Table 2:** Summary of allergenicity and toxicity results of the top 15 candidates from *N. brasiliensis*.

	Allergen prediction					Toxicity prediction
	AllerTOP v. 2.0	AlgPred 2.0. prediction	AlgPred 2.0. IgE Epitopes		SDAP 2.0	ToxinPred 2.0
Protein	Prediction1	Prediction2	No Epitopes	Epitope Type	No allergens	Prediction3
NBR_0000584601	Probable non-allergen	Allergen	0	Non-Allergen	3	Non-Toxin
NBR_0000892601	Probable allergen	Allergen	0	Non-Allergen	5	Non-Toxin
NBR_0001337201	Probable non-allergen	Allergen	1	Allergen	9	Toxin
NBR_0000752401	Probable non-allergen	Allergen	1	Allergen	5	Non-Toxin
NBR_0000897601	Probable non-allergen	Allergen	2	Allergen	0	Toxin
NBR_0001090101	Probable non-allergen	Allergen	0	Non-Allergen	5	Non-Toxin
NBR_0001394301	Probable allergen	Allergen	2	Allergen	39	Toxin
NBR_0000014101	Probable non-allergen	Allergen	1	Allergen	7	Non-Toxin
NBR_0000612601	Probable non-allergen	Allergen	0	Non-Allergen	0	Non-Toxin
NBR_0000723701	Probable allergen	Non-Allergen	1	Allergen	0	Non-Toxin
NBR_0000844701	Probable non-allergen	Non-Allergen	0	Non-Allergen	2	Non-Toxin
NBR_0000992501	Probable non-allergen	Allergen	1	Allergen	0	Non-Toxin
NBR_0000823901	Probable non-allergen	Non-Allergen	1	Allergen	0	Non-Toxin
NBR_0000928101	Probable non-allergen	Non-Allergen	0	Non-Allergen	0	Non-Toxin
NBR_0001356301	Probable non-allergen	Non-Allergen	0	Non-Allergen	4	Toxin

**Table 3:** Summary of allergenicity and toxicity results of the *N. americanus* homologues to the top 15 candidates.

	Allergen prediction					Toxicity prediction
	AllerTOP v. 2.0	AlgPred 2.0. prediction	AlgPred 2.0. IgE Epitopes		SDAP 2.0	ToxinPred 2.0
Protein	Prediction1	Prediction2	No Epitopes	Epitope Type	No allergens	Prediction3
NAME_05148	Probable non-allergen	Allergen	0	Non-Allergen	4	Non-Toxin
NAME_01506	Probable non-allergen	Allergen	0	Non-Allergen	5	Non-Toxin
NAME_06759	Probable non-allergen	Allergen	1	Allergen	0	Non-Toxin
NAME_09716	Probable non-allergen	Allergen	1	Allergen	3	Non-Toxin
NAME_06003	Probable non-allergen	Allergen	0	Non-Allergen	0	Non-Toxin
NAME_05267	Probable non-allergen	Allergen	0	Non-Allergen	5	Non-Toxin
NAME_06878	Probable non-allergen	Allergen	1	Allergen	38	Toxin
NAME_07060	Probable non-allergen	Allergen	0	Non-Allergen	5	Non-Toxin
NAME_01959	Probable non-allergen	Non-Allergen	0	Non-Allergen	0	Non-Toxin
NAME_05186	Probable allergen	Non-Allergen	1	Allergen	0	Non-Toxin
NAME_05865	Probable non-allergen	Non-Allergen	1	Allergen	1	Non-Toxin
NAME_05992	Probable non-allergen	Allergen	0	Non-Allergen	0	Non-Toxin
NAME_03502	Probable non-allergen	Non-Allergen	0	Non-Allergen	0	Non-Toxin
NAME_05701	Probable non-allergen	Non-Allergen	0	Non-Allergen	0	Non-Toxin
NAME_13843	Probable non-allergen	Allergen	0	Non-Allergen	20	Toxin

Complementary analyses showed that eight proteins from both hookworms were stable. Furthermore, regarding protein solubility, five proteins from *N. brasiliensis* (NBR_0000584601, NBR_0000612601, NBR_0000844701, NBR_0000992501, and NBR_0001090101) and eight proteins from *N. americanus* (NAME_05148, NAME_01959, NAME_05865, NAME_05992, NAME_09716, NAME_06878, NAME_07060, and NAME_13843) were predicted as soluble by SoluProt. The top four proteins from each organism in this regard were homologous. Finally, the theoretical isoelectric point was similar in eight of the homologues, differing by less than one point (whole difference range: 0.12–3.1). The difference between four of the remaining candidates was below two points and between two and four points for the last two candidates, which included two acidic proteins (pIs: 5.34 and 5.38) and one highly basic protein (pI: 11.17).

## Discussion

Unlike viral or bacterial vaccinology, the lack of comprehensive parasite-derived omics data has hindered the application of reverse vaccinology approaches for parasitic helminths, particularly for human parasitic nematodes. For the latter, this approach has been limited to the genera *Strongyloides* and *Ascaris* [8, 17]. Following the successful application of an *in silico* vaccine discovery approach to these nematodes, we adapted this analysis to the rodent hookworm *N. brasiliensis*, with the aim of designing appropriate vaccines against the human hookworm *N. americanus*. For this, we followed established and recommended pipelines for *in silico* vaccine discovery comprising four stages: (i) input data gathering and preparation; (ii) prediction of proteins naturally exposed to the immune system; (iii) prediction of epitopes; and (iv) vaccine candidate verification [22]. Cumulative scoring was performed by ranking the different proteins to assign one global score, collectively representing the predicted characteristics without compromising the final selection by one or two weak protein features, unlike other methods, such as a strict filtering workflow [22].

In comparison to the strategy applied to *Strongyloides* spp. and *Ascaris* spp. [8, 17], we observed that the main analyses, though not necessarily in the same order, share commonalities with ours. These include identification of orthologs to study human infections through animal models, analysis of similarity to the host’s proteome for safety concerns, assessment of location (intracellular or extracellular) to identify exposed antigens, immunogenicity prediction based on B and T cell recognition, evaluation of potential allergenicity and physicochemical properties oriented to the production and vaccine formulation. In addition, another important element, especially pertinent for parasites with multi-stage life cycles such as parasitic nematodes, is the analysis of expression by stage, if possible, as described for *Ascaris* spp. [17]. Despite these similarities, the wide array of applications available leads to variations in the inclusion or exclusion of other analyses depending on factors such as the specific pathogen, vaccine format, and researcher criteria. For instance, the studies cited here [8, 17] included other analyses such as predicting transmembrane helices, assessing potential antigenicity by comparing candidates with a curated database, and evaluating glycosylated positions to select epitopes not in contact with these sites. In our analysis, we found differences in several aspects, including the integration of genomic with proteomic data, evaluation of gut location separately from intra/extracellular location, classifying proteins considering predicted functions, and incorporating toxicity and solubility for assessing vaccine safety.

Reverse vaccinology has evolved along with technological advances not only at the wet-lab technical level, allowing the generation of essential omic data, but also in terms of the computational power required to handle constantly growing databases. As capabilities expand, the complexity of these analyses may also increase, but it is essential to balance the diversity of analyses with the relevance of the results to avoid superfluous detail. Considering this and the recent recommendations published by Goodswen *et al.* [22], we consider only the most relevant analyses to design an effective strategy for vaccine candidates prioritization.

### Input data gathering and preparation

One of the first steps in viral or bacterial reverse vaccinology is the identification of conserved regions after aligning the genomes of several strains. However, most hookworm genomes are draft assemblies. Therefore, neither complete genomes nor genomes of other strains are available for intra-species comparison. To overcome this limitation, we approached sequence homology to a closely related hookworm and supported the *in vivo* expression of these proteins using readily available proteomic data.

Our first analysis evaluated the identity (and length) of the predicted proteomes of both hookworms and found that over 12% of the *N. brasiliensis* proteome shared high identity with *N. americanus* proteins in at least 80% of their sequence’s length. In this case, we prioritized proteins showing the highest identity (over 80%) with the highest score to identify the closest functional homologues between these hookworms. However, considering that it is well accepted that proteins sharing over 40% sequence identity are likely to share similar functions, other similarity percentages were also considered and scored [49, 57]. The high similarity between these proteomes has been discussed previously [38]. Importantly, it has recently been demonstrated that although *N. brasiliensis* does not belong to the Ancylostomatidae family, its proteome shares a high degree of similarity (>65% proteins) with *N. americanus* [38], and more than 87% of the proteins secreted by *N. americanus* have a homologue in *N. brasiliensis* [33].

In order to address vaccine safety, potential vaccine candidates should have no significant similarity to mouse or human proteins to avoid the likelihood of inducing an autoimmune response in mammals. Consequently, the evaluation of the homology between the predicted proteome of *N. brasiliensis* and the proteomes of mice and humans assigned values that were inversely correlated with the degree of identity found. Accordingly, despite the minimum length aligned at 40%, we started to deprioritize proteins with an identity as low as 20%, as it is essential to discard any potential structural homology driving immunologically based recognition. More than 55% of the predicted proteins were considered safe in this regard.

### Predicting proteins naturally exposed to the immune system

Proteins identified as located in the extracellular space (including excretory/secretory soluble proteins and extracellular vesicles) are prone to natural exposure to the host immune effector molecules and verified as expressed there at measurable levels. Thus, a higher score in the location analysis was given to proteins previously evidenced by mass spectrometry found in two out of the three compartments evaluated: excretome/secretome of adults or larvae, and extracellular vesicles from adults [16, 54]. Complementarily, since mass spectrometry is not sensitive enough to identify all proteins in a particular sample, some proteins might be expressed by life stages other than those studied (that is, L4 larvae). Thus, we also considered any protein with a predicted signal peptide. This tag is responsible for controlling protein secretion and translocation in all living organisms, and a protein with a signal peptide is thought to be secreted by the organism at some point. A total of 1,669 *N. brasiliensis* proteins satisfied this condition that widened the potential proteins found in the extracellular space. However, considering that the predicted proteome was derived from a draft genome, we cannot discard the possibility that more proteins that meet these criteria could be found in the future.

### Prioritization of candidates by gut location

Most of the pathological effects and morbidity due to hookworm infections result from intestinal blood loss [25]. Additionally, proteins involved in blood digestion and detoxification are crucial for worm establishment and survival [60]. Early results of vaccine candidates based on proteins expressed in the gut of *N. americanus* (*Na*-APR-1 and *Na*-GST-1) have demonstrated an ability to trigger protective humoral responses [1, 65]. These results support the criterion for prioritizing proteins located in the gut of adult hookworms. Considering the importance of gut-expressed candidates, our analysis also included the evaluation of *N. brasiliensis* homologues of molecules identified in a transcriptomic analysis of the gut of *A. ceylanicum* adults by RNA-seq using Illumina sequencing technology [60]. Based on these criteria, only proteins with at least 60% sequence length alignment and at least 30% identity were taken into account. Although *A. ceylanicum* is also a human hookworm, it can also parasitize other hosts, including dogs and cats. This biological distancing is acknowledged in the reduced identity we considered for this analysis when compared with the 40% cutoff we established for comparison with *N. americanus*. In addition, the extended length of alignments with a more flexible identity threshold allows the identification of potential structural homologies that could be transferred into minimal informational predictions for *N. brasiliensis* proteins, providing a starting point for future evaluations.

Our analysis identified 8,920 proteins that shared at least 30% identity with those expressed in the gut of *A. ceylanicum* and, consequently, might also be expressed in the gut of *N. brasiliensis* adults. In this group, 754 proteins were homologues of those found to be upregulated in the gut of *A. ceylanicum* (53 were among the top 100 most expressed proteins) and could be involved in digestion and detoxification. However, further studies aimed at validating these findings should be performed.

### Prioritization of candidates by predicted functions

To identify functions similar to those found in previously identified vaccine candidates [1, 61, 65], we included an additional score based on the domains identified by the Pfam analysis. Functional domain identification provides insights into the potential biological functions of proteins [19] and helps to differentiate functional and folding independent sections within the same protein. Thus, this method provides a means to specifically select a sequence containing the desired functional domain for vaccine design. Hookworm vaccine development is focused on proteins expressed in the adult stage and involved in the digestion of blood, including proteases, transporters, heme-binding, and heme-detoxification proteins [31, 34, 60]. In addition, proteins in direct contact with blood are more likely to be neutralized by host antibodies [31].

Hemoglobin digestion is performed sequentially using at least three different enzymes. *Na*-APR-1 assists in breaking down hemoglobin released from ruptured erythrocytes, whereas *Na*-CP-3 and *Na*-MEP-1 only cleave globin fragments released by hydrolysis with *Na*-APR-1. The final process for the release of amino acids is performed by exopeptidases. Accordingly, the prioritized group of proteins comprises aspartyl (APR), cysteinyl (CP), and metalloproteases (MEP) that have been identified in the intestinal brush border of *N. americanus* [31]. One-fourth of the proteins prioritized by Pfam domains (score ≥1) were predicted to have a domain implicated in parasite feeding, such as proteases, peroxidases, and peptidases, which are crucial for hemoglobin digestion and parasite survival within the host.

Interestingly, compared to other hookworms, the ES of *N. americanus* includes a higher abundance of aspartyl proteases because they are implicated not only in parasite feeding, but also in skin penetration and host tissue degradation [33]. Owing to its vital role, *Na*-APR-1, in combination with *Na*-GST-1, has been selected for human vaccination in ongoing clinical trials [1, 65]. Remarkably, the present computational filtering retrieved three *Nb*-APRs among the top 15 candidates, which is consistent with the selection of *Na*-APR-1 for ongoing clinical trials.

Moreover, *Na*-GST-1 is known for heme detoxification through the prevention of oxidative damage and is widely distributed within parasitic tissues, including the cuticle/hypodermis, muscle, gut, and esophagus. Other functions, such as the acquisition of exogenous heme and related compounds or host immune modulation, are under investigation [63]. Despite its potential, we did not prioritize this function, considering that its expression is not restricted to the adult stage, nor is it limited to the gut, which may induce IgE-driven responses in previously exposed individuals. As shown with the vaccine candidate *Na*-ASP-2, this undesirable allergenic response limits their use because of the generalized urticarial reactions triggered in some volunteers [12].

Other important proteases implicated in parasitic feeding include cysteine and metalloproteases. Cysteine proteases are upregulated during the transition from free-living larvae to blood-feeding adult worms, indicating their important role in nutrient acquisition. In addition to its proteolytic activity against hemoglobin, proteolysis against antibodies and fibrinogen has also been described [33]. Similarly, metalloproteases have been associated not only with parasite feeding, but also with migration and invasion through human tissue. For instance, a number of astacin metalloproteases were upregulated in larvae and reported to inhibit eosinophil recruitment by cleaving eotaxin, a potent eosinophil chemoattractant [33]. Considering our computational scoring, one cysteine protease and seven metalloproteases were found among the top-50 candidates, making a total of 11 candidates associated with parasite feeding and other important functions among the top 50 out of the 22,796 proteins analyzed.

### Predicting epitopes

The ultimate objective of vaccine design is to trigger an effective immune response that can eliminate pathogens and re-establish host homeostasis. Thus, an effective vaccine formula must combine pathogen-derived components, such as epitopes, with an appropriate adjuvant to induce a strong Th2 response accompanied by the production of neutralizing antibodies that can bind complement proteins, especially C3b, to increase parasite opsonization [61]. These traits were evaluated by an immunoinformatic analysis that considered not only the identification of epitopes with the potential to be presented by the HLA-II supertypes (NetMHCIIpan 4.0), but also to be recognized by specific antibodies (BepiPred 3.0).

It is important to emphasize that, although it is believed that approximately 90% of the epitopes of native antigens are discontinuous (conformational) epitopes, the epitopic residues identified in our study are considered only for continuous (or linear) epitopes. The reason was that the prediction quality of discontinuous epitopes is not homogeneous at a proteome level yet. Although the three-dimensional structure has been experimentally resolved for only a fraction of protein, this pitfall have been reasonably addressed by recent *ab initio* structural predictors such as AlphaFold 2.0 [30]. However, the involvement of this considerable gradient in the structural quality in conformational epitope prediction remains to be analyzed in detail. Thus, we utilized a state-of-the-art method for the prediction of linear epitopes, BepiPred 3.0, that utilizes natural processing language and transfer learning at the whole-protein level. By doing so, developers claim that the method can capture structural level epitopic traits and perform similarly to discontinuous epitope predictors [7]. Of the identified epitopic proteins, 35% displayed more than 120 residues in their amino acid chains. Considering B-cell epitopes as fragments composed of nine or more consecutive residues, top-scoring proteins may display over ten potential B-cell epitopes with the ability to bind antibodies to neutralize the antigen and/or initiate the complement cascade [45].

In addition, NetMHCIIpan 4.0 can find epitopes with high binding affinity and broad population protection coverage. Predicting peptide binding affinities to HLA class-II molecules is more challenging than that of HLA-I molecules, those involved in the cellular cytotoxic response, considering the higher polymorphic nature of HLA class-II molecules, variations in peptide length, influence of peptide flanking regions, and increased difficulty in identifying the proper peptide-binding core. Although characterizing the peptide binding profiles of all existing HLA class-II alleles is not feasible, extensive improvements have been applied to the NetMHCIIpan 4.0 algorithm with a dataset 2.5 times bigger and more than twice the number of alleles [29]. In NetMHCIIpan 4.0, the combined allele frequency of the set of 80 HLA class-II molecules of three loci (36 HLA-DR, 27 HLA-DQ, 9 HLA-DP, and 8 mouse MHC class-II) resulted in >99% population coverage [59].

In addition, the difficulty in analyzing population coverage, given the huge variety of HLA molecules and their differential expression depending on ethnic groups, has been overcome by establishing HLA supertypes. HLA supertypes are defined by the clustering of HLA alleles depending on their ability to bind largely overlapping sets of epitopes [32]. Therefore, a variety of genetic backgrounds can be represented by a set of representative HLA alleles with abundant supertypes in the target population.

The complete set of supertypes used to analyze the population coverage presented a potential global coverage of 97.48%, as retrieved by the IEDB PPC tool [5]. Therefore, a vaccine formulation comprising this set of HLA-II supertypes in one antigen or through the complementary combination of several candidates has the potential to offer effective global population coverage [21]. Considering the wide distribution of hookworm infections, it would be ideal for a vaccine to cover this set of HLA class-II subtypes. The NetMHCIIpan 4.0 analysis of the *N. brasiliensis* proteome provided a set of 1,904 proteins with the potential to be presented to one or more HLA class-II supertypes with high affinity and thus to be strategically combined. Similarly, for the transfer of mouse vaccinology results to their human counterparts, the rational selection of complementary candidates will potentially achieve the target population coverage.

### Vaccine candidate verification

The final scoring of the *N. brasiliensis* predicted proteome (Supplementary Table 8) relied on different synergistic analyses. Hence, the top-scoring proteins did not necessarily receive the highest score in all the evaluated traits; however, this computational analysis provides a comprehensive understanding of the importance of these proteins, not only from an immunological perspective, but also from a functional perspective and helps narrow down the selection of potential vaccine candidates. It is worth mentioning that the top 15 sets of proteins included those closely related to vaccine candidates that have been shown to be effective in inducing humoral responses and avoiding undesired generalized urticaria in pre-exposed humans, which highlights the suitability of our approach. At the same time, all these proteins have been validated by proteomic analysis in at least one of the extracellular spaces of adults or larvae. Therefore, these proteins potentially play crucial roles in larvae and adults during their life cycle and are key at the interface contact between the host and parasites.

### Allergenicity, toxicity, and physicochemical properties of top-15 candidates

One of the challenges in hookworm vaccinology is the identification of immunogenic proteins that can trigger effective humoral responses, even in pre-exposed individuals, without the problem of generalized urticaria caused by the presence of IgE antibodies, similar to vaccination of pre-exposed adults with *Na*-ASP-2 protein [12]. Therefore, an allergenicity analysis was performed to identify potential allergenic epitopes within the structures of the top candidates. Like many other immunogenic tests, many algorithms have been designed for the analysis of allergenicity. Three of these, AllerTOP V.2.0, AlgPred, and SDAP 2.0, were selected to provide considerable data for the complete consensus interpretation of the allergenicity results for *N. brasiliensis* and its homologues in *N. americanus* (Supplementary Tables 9 and 10, respectively) prior to use in vaccine formulations. Since the human allergenicity analysis retrieved diverse results depending on the program applied, including potential false positives, we only consider “strong computational evidence” when the three programs retrieved similar results; however, it is important to experimentally validate the potential allergic responses using *in vivo* or *in vitro* tests to discard potential harmful effects.

Additionally, as recommended by Goodswen *et al.* in the last “reverse vaccinology guide” [22], we estimated toxicity, solubility, and other physicochemical characteristics that would be useful for the evaluation of potential harmful effects, optimization of protein production, and vaccine formulation. When toxicity was compared, only two of the four *N. brasiliensis* proteins were predicted to be toxic in *N. americanus* homologs, and thus, will require experimental testing to confirm or discard such predicted damaging capacity. Furthermore, the solubility and stability substantially differed in approximately half of the candidates. This may reflect the differences in the corresponding hosts, as well as the need for further experimental evaluations to optimize protein production and vaccine formulation.

## Conclusions

Here, we present the first application of an *in silico* vaccine discovery approach for hookworms. These parasitic nematodes continue to represent a human health burden, affecting to some extent up to one-sixth of the world’s population. This bioinformatic-based approach enhances the discoverability of vaccine candidates. This study combines expertise in hookworm biology with the high-throughput performance of current computational tools to prioritize potential vaccinable antigenic proteins, thus opening the path to experimental validation. Additionally, it provides valuable data for the rational design of a multifunctional and multiantigenic formulation, able to trigger cellular and humoral immunity, with the potential to provide the broad coverage required. The computational workflow was strictly adapted to the pathogen of interest and balanced the inclusion of meaningful evaluations. Therefore, despite the availability of further theoretical evaluations, these parameters mainly rely on the final vaccine formulation, including the selection of an appropriate adjuvant that will be experimentally defined.

Taken together, our *in silico* evaluations have predicted a group of immunogenic proteins with high homology to their human hookworm counterparts and with several advantages, as they provide greater safety and reduced allergenic effects compared to the live attenuated vaccine. Furthermore, our analysis considers the excretome/secretome of adults and larvae and, thus, has the potential to cover the key stages in host-parasite interactions. However, the present study is a purely *in silico* computational approach. Accordingly, it has several limitations including the lack of experimental validation and animal-based determination of their efficacy in antiparasitic prophylaxis. Therefore, *in vitro* and *in vivo* validation of the immunogenicity of the potential vaccine candidates should be performed before they can be assessed in vaccination programs.

## Supplementary material

The supplementary material for this article can be found at https://www.parasite-journal.org/10.1051/parasite/2025025/olm. *Supplementary Table 1*: Data sheet containing all the results from the evaluation of the identity of the predicted proteomes of *Nippostrongylus brasiliensis* with *Necator americanus*. Nam_ident: Identity percentage with *Necator americanus*. Sc_Nam: score assigned to each of the *N. brasiliensis* proteins. *Supplementary Table 2*: Data sheet containing all the results from the protein homology results between *Nippostrongylus brasiliensis* and mammalian species. Hsa_ident: Percentage of identity with *Homo sapiens*; Mmu_ident: percentage of identity with *Mus musculus*; Sc_Mammal: score assigned to each protein based on homology with other mammals; NF: not found. *Supplementary Table 3*: Data sheet containing all the results from extracellular localization. Excreted secreted products (ES) or extracellular vesicles (EVs) secreted by adult or L3 larvae. *Supplementary Table 4*: Data sheet containing all the results from the analysis of *Nippostrongylus brasiliensis* proteins with homologs expressed in the gut of the related hookworm *Ancylostoma ceylanicum.* *Supplementary Table 5*: Data sheet containing all the results from the Pfam-based analysis that allocated *Nippostrongylus brasiliensis* proteins into four different groups according to the domain families to which they belonged. *Supplementary Table 6*: Data sheet containing the results of the identification of linear B-cell epitopes and score given to each *Nippostrongylus brasiliensis* protein. Bepit_res: number of B-cell epitope residues; Bepit_res_perc: percentage of B-cell epitope residues compared to the total number of protein residues. *Supplementary Table 7*: Data sheet containing the results of the identification of the presence of HLA class II epitopes with extended population coverage and score given to each *Nippostrongylus brasiliensis* protein. *Supplementary Table 8*: Data sheet summarising all the results from each evaluation applied to the *Nippostrongylus brasiliensis* predicted proteins. Proteins are ranked by total score. *Supplementary Table 9*: Data sheet containing a summarized and extended table of allergenicity and toxicity results of the top-15 candidates from *Nippostrongylus brasiliensis*. *Supplementary Table 10*: Data sheet containing a summarized and extended table of the solubility and physicochemical properties of the top-15 candidates from *Nippostrongylus brasiliensis*. *Supplementary Table 11*: Data sheet containing a summarized and an extended table of the allergenicity and toxicity results of the top-15 candidate homologs in *Necator americanus.* *Supplementary Table 12*: Data sheet containing a summarized and extended table of the solubility and physicochemical properties of the top-15 candidate homologs in *Necator americanus*.
